# Ovarian Carcinosarcoma With Heterologous Chondrosarcomatous Differentiation: A Case Report and Review of Literature

**DOI:** 10.7759/cureus.110975

**Published:** 2026-06-16

**Authors:** Amal Moukhtari, Asmae Aissaoui, Anass Haloui, Nassira Karich, Amal Bennani

**Affiliations:** 1 Department of Pathology and Cytology, Centre Hospitalo-Universitaire Mohammed VI d'Oujda, Oujda, MAR; 2 Department of Pathology, Mohammed VI University Hospital, Faculty of Medicine, Mohammed 1st University, Oujda, MAR; 3 Department of Anatomopathology, Mohammed VI University Hospital, Faculty of Medicine, Mohammed 1st University, Oujda, MAR

**Keywords:** chondrosarcoma, high-grade serous carcinoma, immunohistochemistry, malignant mixed müllerian tumor, ovarian carcinosarcoma, peritoneal carcinomatosis

## Abstract

Ovarian carcinosarcoma is a rare and aggressive malignancy characterized by the coexistence of epithelial and mesenchymal components. It poses considerable diagnostic and therapeutic difficulties largely due to its high propensity for peritoneal dissemination and unfavorable prognosis.

We report the case of a 58-year-old woman presenting with progressive abdominal distension and pelvic pain. Imaging revealed a left ovarian mass associated with ascites and diffuse peritoneal carcinomatosis.

Cytological analysis of ascitic fluid demonstrated malignant cells and histopathological examination of omental and peritoneal biopsies showed a biphasic tumor, composed of a high-grade serous carcinoma component and a heterologous chondrosarcomatous component.

Immunohistochemistry revealed positivity for CK7, WT1, P16, and diffuse p53 expression, supporting a high-grade serous epithelial origin, while CK20 was negative.

The diagnosis of ovarian carcinosarcoma with heterologous differentiation was retained. Given the advanced stage, the patient was considered for platinum-based chemotherapy.

This case highlights the diagnostic challenges and histopathological features of ovarian carcinosarcoma.

The current therapeutic regimen for carcinosarcoma is still based on tumor cytoreduction and platinum-containing chemotherapy; research on targeted therapy is still in progress.

## Introduction

Ovarian carcinosarcoma, also known as malignant mixed Müllerian tumor, is a rare neoplasm characterized by a biphasic histological pattern combining a carcinomatous (epithelial) component and a sarcomatous (mesenchymal) component. These tumors are highly aggressive, are often diagnosed at an advanced stage with peritoneal dissemination, and recur within one year after the end of the initial treatment in most patients. Recent molecular studies suggest a monoclonal origin, supporting the hypothesis that the sarcomatous component arises through dedifferentiation of the epithelial carcinoma, most commonly of high-grade serous type. Heterologous elements, such as chondrosarcoma, osteosarcoma, or rhabdomyosarcoma, may be present and are associated with a worse prognosis.

Despite its rarity, ovarian carcinosarcoma remains one of the most aggressive gynecologic malignancies, frequently presenting at an advanced stage and carrying a poor prognosis. Patients commonly present with nonspecific symptoms such as abdominal distension, pelvic pain, ascites, and a pelvic mass, which may complicate early diagnosis. Radiological findings are often non-specific, and definitive diagnosis relies on histopathological and immunohistochemical evaluation. Current treatment strategies are largely extrapolated from those used for high-grade epithelial ovarian carcinoma and generally include cytoreductive surgery and platinum-based chemotherapy. Nevertheless, the optimal management and prognostic significance of several pathological features remain incompletely understood because of the rarity of the disease. Heterologous chondrosarcomatous differentiation represents an uncommon histological finding that may create diagnostic challenges and reflects the remarkable morphological heterogeneity of these tumors. Reporting such cases contributes to a better understanding of their clinicopathological spectrum and may help refine diagnostic and therapeutic approaches in this rare malignancy.

We report a case of ovarian carcinosarcoma with heterologous chondrosarcomatous differentiation, highlighting its clinicopathological features and reviewing the current literature.

## Case presentation

A 58-year-old postmenopausal woman, nulligravid and nulliparous, with no significant past medical, surgical or gynecological history and no family history of ovarian, breast endometrial, or other malignancies presented with a one-month history of progressive abdominal distension and hypogastric pain as well as nausea, clinical examination revealed a distended abdomen with shifting dullness suggestive of ascites and mild hypogastric tenderness, rectal examination was unremarkable, no peripheral lymphadenopathy was noted.

Laboratory tests revealed elevated CA125 levels at 235 U/mL (reference range: <35 U/mL) and thrombocytosis with a platelet count of 511,000/mm³ (reference range: 150,000-400,000/mm³). The remaining laboratory parameters were within normal limits.

Abdominal ultrasound revealed a moderate amount of intraperitoneal fluid consistent with ascites (Figure 1), along with a pelvic mass (Figure 2) in addition to peritoneal carcinosis (Figure 3). Meanwhile, the liver was normal in size with regular contours, although a perihepatic fluid collection was noted; other abdominal organs, including the pancreas, spleen, and kidneys, appeared unremarkable. 

**Figure 1:**
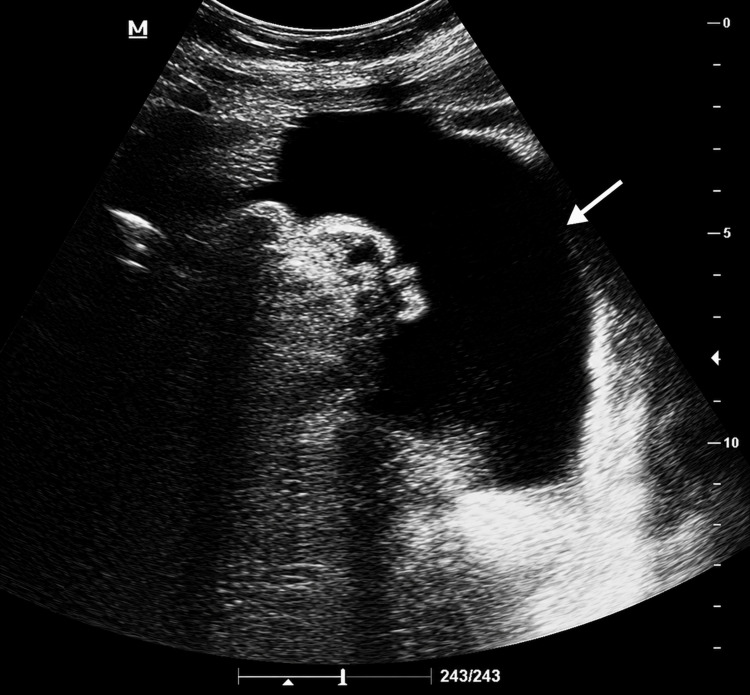
Abdominal ultrasound showing large-volume ascites. Ultrasound images demonstrate abundant anechoic intraperitoneal fluid surrounding abdominal organs, consistent with significant ascites, with internal echogenic structures suggestive of peritoneal tumor implants. The white arrow highlights the ascitic collection.

**Figure 2:**
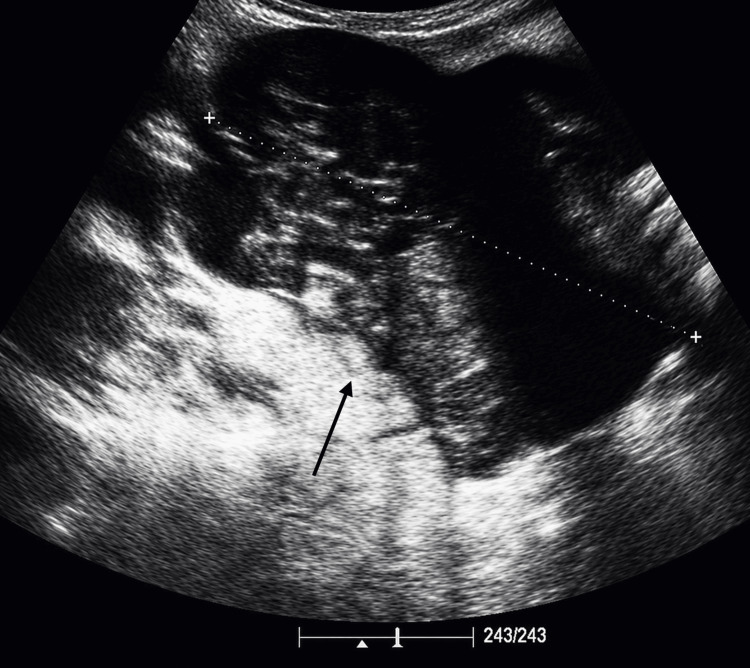
Ultrasound image of a large pelvic mass. A heterogeneous pelvic mass with mixed echogenicity is identified, showing both solid and cystic components. The lesion appears poorly circumscribed. The black arrow highlights the ovarian tumor.

**Figure 3:**
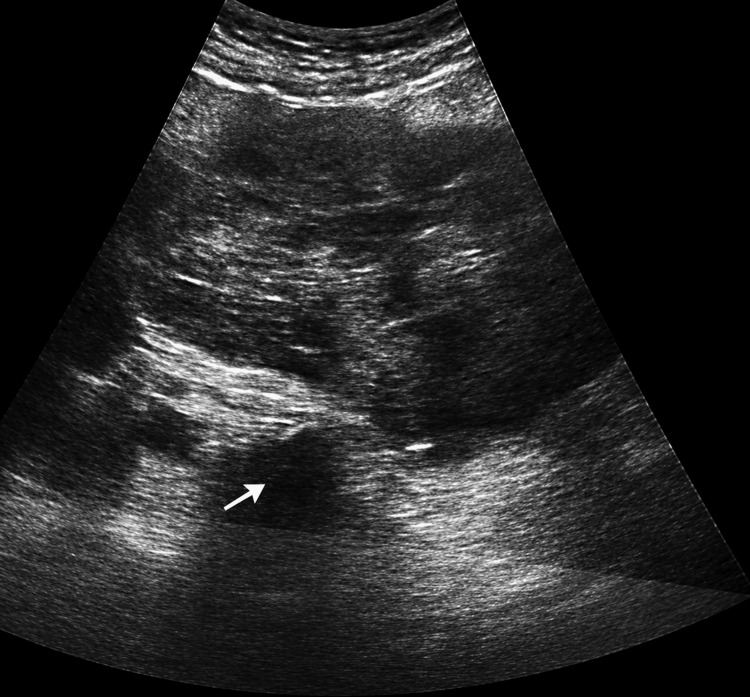
Ultrasound features suggestive of peritoneal carcinomatosis. Irregular echogenic thickening and nodular lesions within the peritoneal cavity and omental fat are observed, consistent with peritoneal tumor implants (omental cake); the white arrow highlights the carcinosis.

Contrast-enhanced computed tomography (CECT) of the abdomen and pelvis demonstrated large-volume ascites associated with diffuse peritoneal and omental involvement, characterized by multiple nodular lesions within the mesentery and epiploic regions. The largest peritoneal nodule, located in the supra-digestive region, measured approximately 38 × 28 mm (Figure 4), along with a large left adnexal mass measuring approximately 120 × 105 mm, with solid and cystic components and heterogeneous contrast enhancement, suggestive of a malignant ovarian tumor (Figures 5, 6). The liver, spleen, pancreas, kidneys, and major vascular structures were unremarkable, with no evidence of focal hepatic lesions or vascular abnormalities. No significant lymphadenopathy was identified. There was no evidence of distant organ metastasis.

**Figure 4:**
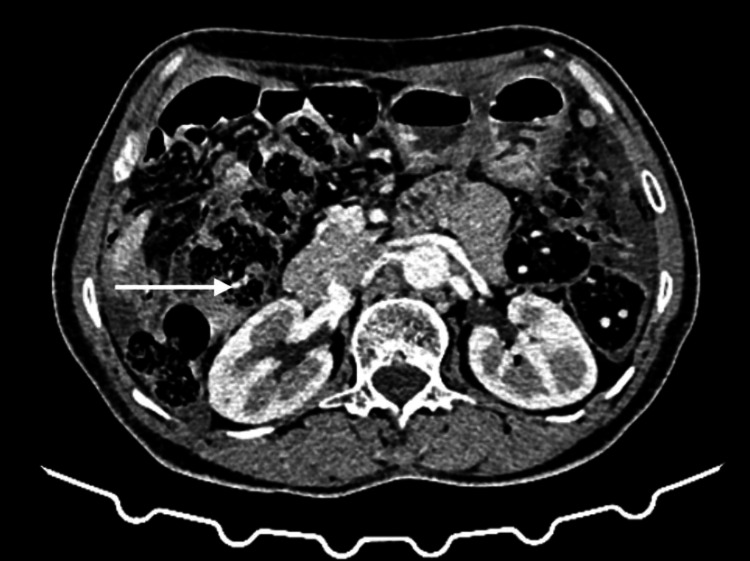
Axial contrast-enhanced CT scan showing peritoneal carcinomatosis. Axial contrast-enhanced abdominal CT image demonstrates diffuse irregular nodular thickening of the omentum and mesenteric fat infiltration, producing an “omental cake” appearance suggestive of peritoneal carcinomatosis. Associated bowel loop crowding is also observed. The white arrow highlights the omental thickening.

**Figure 5:**
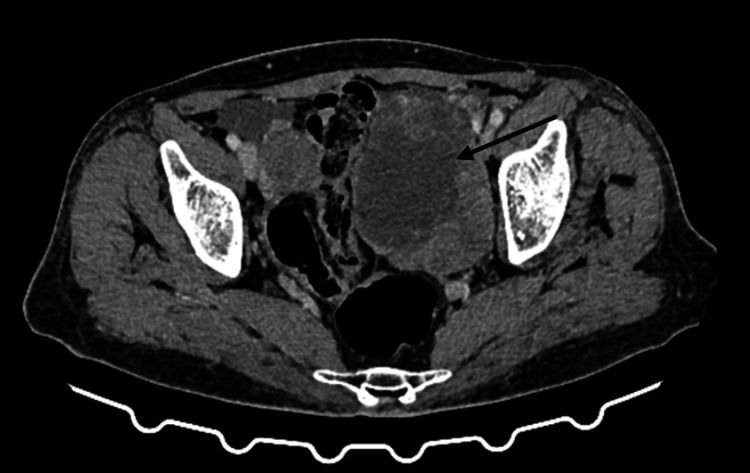
Axial contrast-enhanced CT scan showing a pelvic ovarian mass. Axial pelvic CT image demonstrates a large heterogeneous left adnexal mass with predominantly soft-tissue attenuation, highly suggestive of an ovarian neoplasm. The black arrow highlights the ovarian mass exerting a mass effect on adjacent pelvic structures.

**Figure 6:**
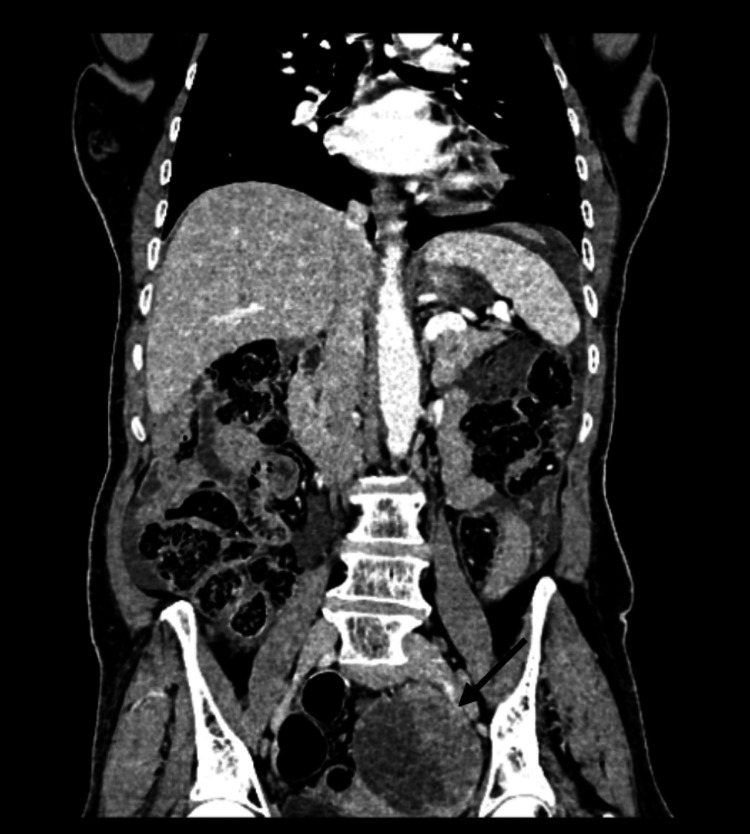
Coronal computed tomography CT image demonstrating the extent of the ovarian tumor. Coronal abdominal-pelvic computed tomography CT reconstruction reveals a voluminous heterogeneous pelvic mass arising from the left ovary, associated with displacement of adjacent bowel loops and extension into the pelvic cavity. The black arrow indicates the ovarian mass.

Overall, the imaging findings were highly suggestive of a primary left ovarian malignancy associated with diffuse peritoneal carcinomatosis and massive ascites. Based on the presence of extensive peritoneal involvement beyond the pelvis demonstrated on imaging and confirmed by omental and peritoneal biopsies, the disease was classified as International Federation of Gynecology and Obstetrics (FIGO) stage IIIC ovarian cancer.

Paracentesis was performed, and cytological analysis of ascitic fluid showed numerous highly atypical malignant cells.

To establish a definitive diagnosis, the patient underwent exploratory surgery with omental and parietal peritoneal biopsies. Histological examination revealed a malignant biphasic proliferation involving fibrofatty tissue; the epithelial component consisted of a high-grade carcinoma with papillary, solid, and nested architecture. Tumor cells exhibited marked nuclear atypia, pleomorphism, prominent nucleoli, and abundant eosinophilic cytoplasm. Numerous mitotic figures were identified (>12 mitoses per 10 high-power fields). Psammoma bodies were also observed (Figures 7, 8). The mesenchymal component showed areas of lobulated structures composed of pleomorphic cells with hyperchromatic nuclei and vacuolated cytoplasm embedded in a chondro-myxoid stroma, consistent with chondrosarcomatous differentiation (Figures 9-11). Vascular emboli and perineural invasion were identified.

**Figure 7:**
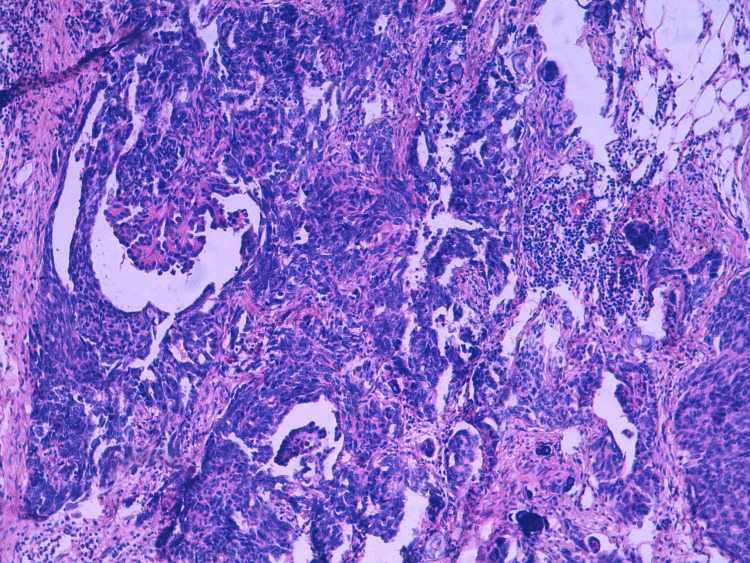
Papillary structures within the epithelial component of the tumor (hematoxylin and eosin stain, x200).

**Figure 8:**
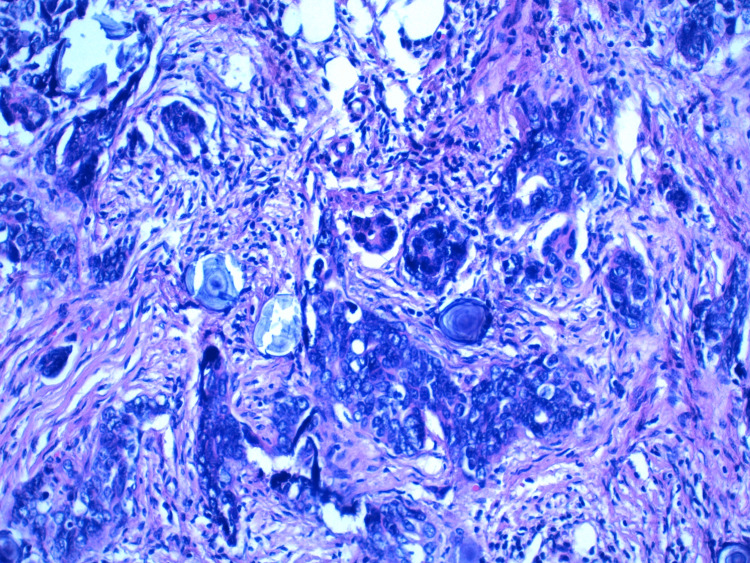
The epithelial component of the tumor consistent with high-grade serous carcinoma (hematoxylin and eosin stain, ×200). The neoplastic cells are arranged in complex papillary structures with marked nuclear pleomorphism and hyperchromasia. Numerous psammoma bodies are present.

**Figure 9:**
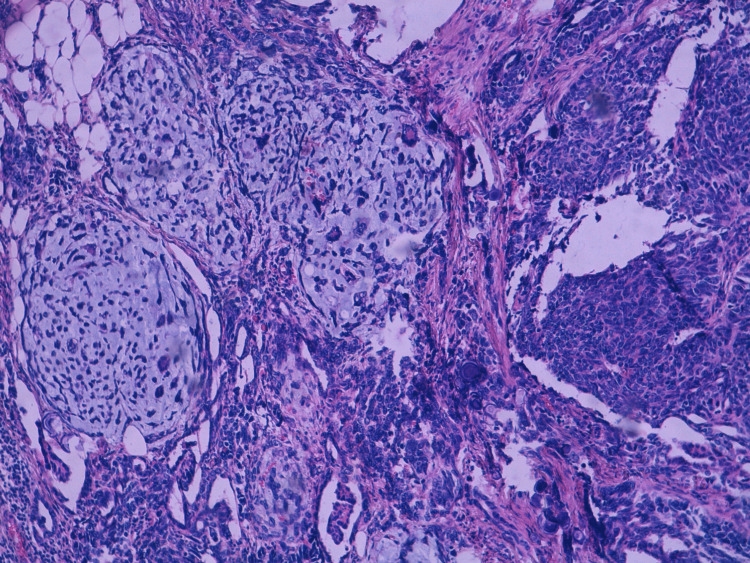
Histopathological examination showing the biphasic nature of the tumor with juxtaposition of epithelial and mesenchymal components (hematoxylin and eosin stain, ×200). The epithelial component displays malignant glandular structures, while the adjacent mesenchymal component is composed of atypical spindle cells. Areas of heterologous chondrosarcomatous differentiation are also identified.

**Figure 10:**
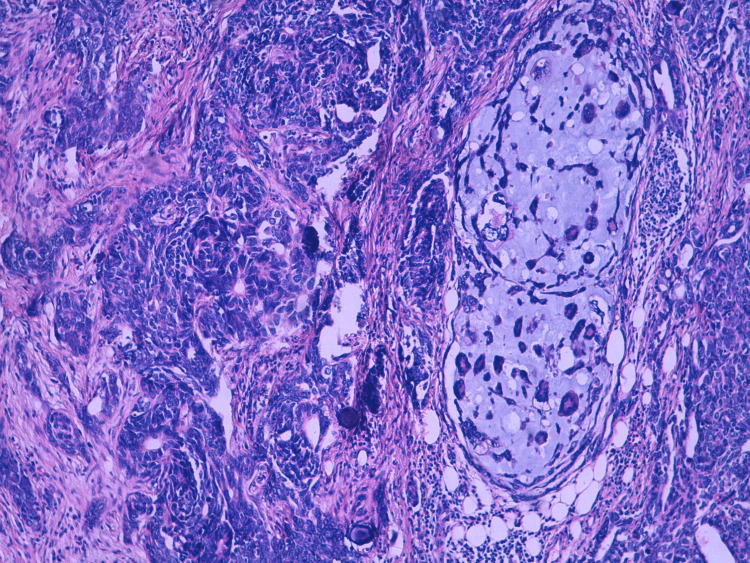
Histopathological examination showing heterologous chondrosarcomatous differentiation within the ovarian carcinosarcoma (hematoxylin and eosin stain, ×200). The image demonstrates a well-defined cartilaginous matrix containing atypical chondrocytes embedded in lacunae, associated with scattered multinucleated giant cells, consistent with chondrosarcomatous differentiation.

**Figure 11:**
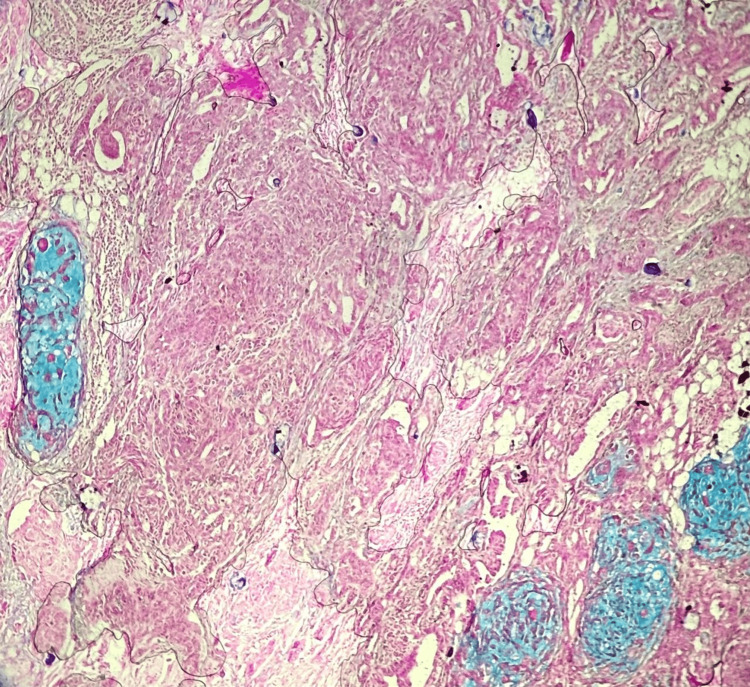
Alcian blue stain highlighting a prominent chondromyxoid extracellular matrix (×100), consistent with heterologous chondrosarcomatous differentiation.

Immunohistochemical analysis demonstrated diffuse positivity for CK7 (Figure 12) and WT1 (Figure 13), strong and diffuse p53 expression (Figure 14), focal and heterogeneous expression of estrogen receptor (ER), diffuse and strong expression of p16 (Figure 15), and negative staining for CK20 and progesterone receptor (PR).

**Figure 12:**
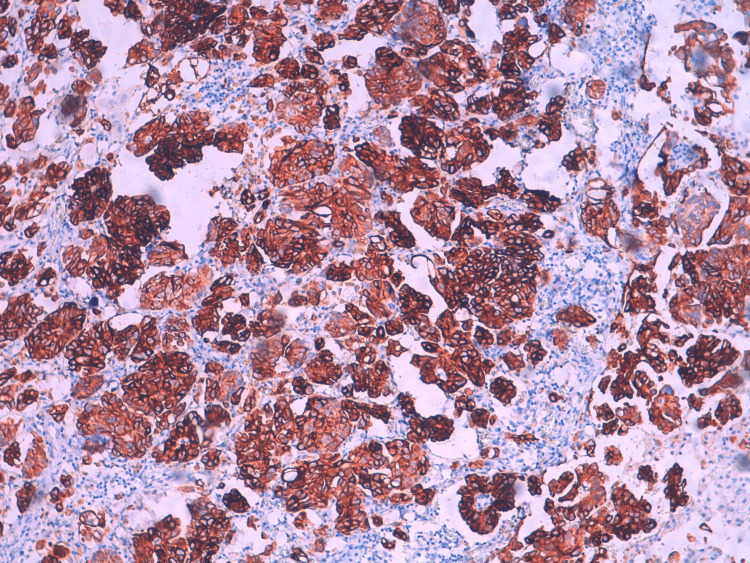
Immunohistochemical staining shows diffuse and strong cytoplasmic expression of CK7 in tumor cells (CK7 immunostain, ×200), highlighting the epithelial component

**Figure 13:**
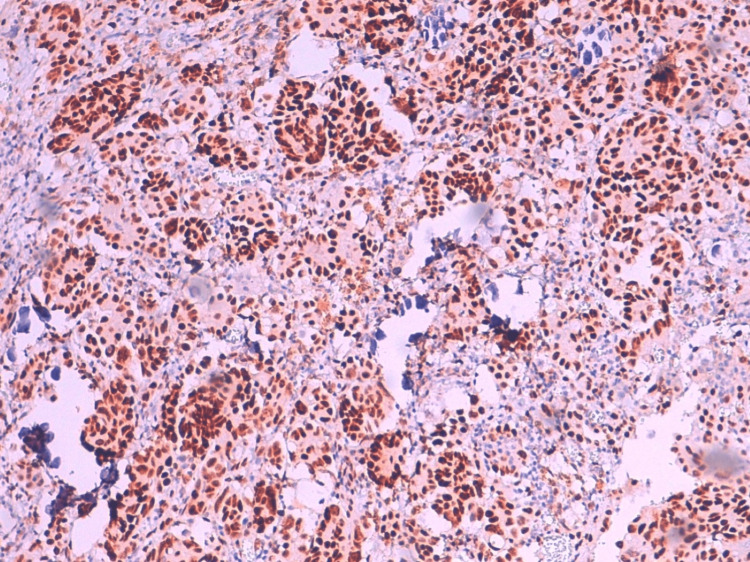
Immunohistochemical staining showing diffuse and strong nuclear expression of WT1 in the epithelial component of the tumor (WT1 immunostain, ×200), supporting high-grade serous differentiation.

**Figure 14:**
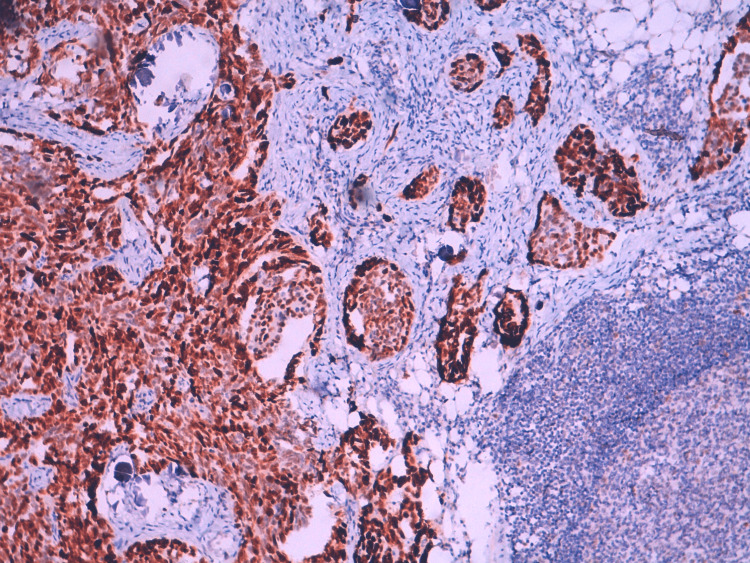
Immunohistochemical staining shows diffuse and strong nuclear overexpression of p53 in tumor cells (p53 immunostain, ×200), consistent with a mutant TP53 pattern.

**Figure 15:**
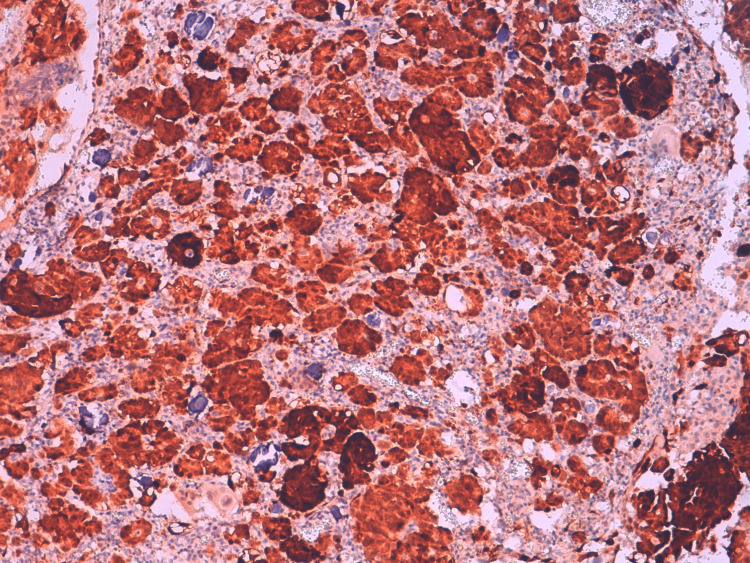
Immunohistochemical staining showing diffuse and strong (“block-type”) expression of p16 in tumor cells (p16 immunostain, ×200), supporting high-grade serous carcinoma differentiation.

PAX8 showed positive nuclear staining in the epithelial component, supporting Müllerian differentiation and a primary ovarian origin.

The sarcomatous component showed a positive expression of CK7 and p53, with negative staining for CK20, WT1, RE, and RP.

The histopathological features aligned with the clinical presentation and imaging studies confirm the aggressive nature of the lesion. The tumor corresponds to an ovarian carcinosarcoma composed of a high-grade serous carcinoma and a heterologous chondrosarcomatous component, with vascular and perineural invasion.

The case was subsequently discussed at a multidisciplinary tumor board meeting, and given the extent of peritoneal disease, neoadjuvant carboplatin-paclitaxel chemotherapy was initiated. The patient is currently receiving treatment and remains under close oncological follow-up. Reassessment imaging is planned after completion of chemotherapy to evaluate tumor response and determine the feasibility of interval cytoreductive surgery.

## Discussion

Ovarian carcinosarcoma is a rare and highly aggressive malignancy, accounting for less than 5% of ovarian cancers. Patients often present with nonspecific symptoms including abdominal mass, abdominal distension, abdominal pain, ascites, occasional vaginal bleeding, and nonspecific gastrointestinal symptoms, making early diagnosis challenging. Advanced-stage presentation (FIGO stage III or IV) is common as illustrated in our case [1-5]. The metastatic pattern of ovarian carcinosarcoma is not fully understood but appears to involve multiple routes, including direct extension, peritoneal dissemination, and lymphatic spread, similar to other ovarian malignancies. High rates of lymph node involvement and vascular invasion have been reported, with many patients presenting with extra-ovarian disease and associated ascites. In our case, peritoneal biopsy confirmed metastatic involvement, along with omental carcinomatosis.

Several hypotheses have been proposed to explain the histogenesis of carcinosarcoma. Among these, the transformation (or metaplastic) theory suggests that the sarcomatous component arises from the epithelial carcinoma during tumor progression. Another widely accepted concept is the monoclonal origin theory, which proposes that both epithelial and mesenchymal components derive from a single pluripotent progenitor cell that undergoes divergent differentiation early in tumor development. In contrast, the collision theory postulates that the carcinomatous and sarcomatous elements originate independently from distinct cell populations that subsequently merge. Recent molecular and immunohistochemical studies strongly support a monoclonal origin, whereby the sarcomatous component arises through dedifferentiation or epithelial-mesenchymal transition (EMT) from the epithelial carcinoma [2]. Recent genomic analyses have demonstrated shared molecular alterations between the epithelial and sarcomatous components, including TP53 mutations and chromosomal instability patterns, further supporting a monoclonal origin and the pivotal role of EMT in tumor progression and heterologous differentiation [6,7].

This theory is reinforced by the presence of shared genetic alterations, particularly TP53 mutations, and overlapping immunophenotypic profiles between both components. In our case, the diffuse p53 expression and WT1 positivity are consistent with a high-grade serous carcinoma origin, which represents the most common epithelial component in ovarian carcinosarcomas. Histopathological examination remains essential for diagnosis. Carcinosarcoma is characterized by the coexistence of epithelial and mesenchymal components. The epithelial component may exhibit various histological subtypes, including endometrioid, serous, or clear cell carcinoma, typically arranged in glandular, nested, or solid patterns. In the present case, a high-grade serous component was identified. The mesenchymal component may consist of either homologous or heterologous elements, such as endometrial stromal sarcoma, leiomyosarcoma, chondrosarcoma, osteosarcoma, rhabdomyosarcoma, or liposarcoma. In our case, the sarcomatous component was represented by chondrosarcoma, a relatively uncommon heterologous element in ovarian carcinosarcoma. Chondrosarcomatous differentiation is noteworthy because it reflects the remarkable plasticity of these tumors and provides further evidence of divergent mesenchymal differentiation. Several studies have suggested that heterologous elements may correlate with increased biological aggressiveness, although their independent prognostic significance remains controversial [6-8]. Histologically, the identification of a chondro-myxoid matrix highlighted by Alcian blue staining further supports this differentiation. The coexistence of vascular emboli and perineural invasion in our case also reflects the highly invasive nature of this tumor.

From a diagnostic perspective, ovarian carcinosarcoma may pose challenges, particularly in limited biopsy samples. The differential diagnosis includes high-grade serous carcinoma with sarcomatoid features, metastatic carcinosarcoma, and primary peritoneal carcinoma [2,4]. In this context, although the histological diagnosis was established on omental and peritoneal biopsies, the diagnosis of primary ovarian carcinosarcoma was supported by the presence of a large unilateral left adnexal mass on imaging, elevated CA-125 levels, diffuse WT1 positivity, CK7 positivity, and positive PAX8 expression, all consistent with a Müllerian ovarian primary, while the absence of CK20 helps exclude a gastrointestinal primary [2,9].

Immature teratoma is another important differential diagnosis, particularly in tumors showing heterologous elements. However, it typically occurs in younger patients and is characterized by the presence of immature embryonal tissues derived from germ cell layers, especially neuroectodermal differentiation.

Therapeutically, despite its sarcomatous component, ovarian carcinosarcoma is currently managed in a manner similar to high-grade epithelial ovarian carcinoma. According to the National Comprehensive Cancer Network (NCCN) and the European Society for Medical Oncology (ESMO) guidelines, platinum-based chemotherapy remains the standard of care [10-17]. The combination of carboplatin and paclitaxel has demonstrated improved outcomes and better tolerability compared to older regimens such as ifosfamide-based therapies [17]. This reflects the biological behavior of these tumors, which is predominantly driven by the epithelial component.

Surgical management also plays a central role in treatment. Maximal cytoreductive surgery is considered a key prognostic factor, with several retrospective studies demonstrating improved survival in patients who achieve optimal tumor debulking. Optimal cytoreduction is generally defined by the presence of minimal residual disease, commonly less than 1 cm in maximal diameter following surgery.

Emerging data suggest that targeted therapies may have a role in selected patients. Given the molecular similarities with high-grade serous carcinoma (HGSC), including frequent TP53 mutations and potential homologous recombination deficiency (HRD), the use of poly (ADP-ribose) polymerase (PARP) inhibitors as maintenance therapy may be considered, particularly in patients harboring BRCA1/2 mutations or HRD-positive tumors [10,13,18]. Additionally, anti-angiogenic agents such as bevacizumab may be beneficial in advanced-stage disease with peritoneal carcinomatosis.

Despite these therapeutic advances, the prognosis of ovarian carcinosarcoma remains poor, with lower survival rates compared to conventional high-grade serous carcinoma [5]. Several factors have been shown to influence patient outcomes, including clinical stage at diagnosis, presence of heterologous elements, the extent of residual disease following surgery, serum CA-125 levels, and the type of chemotherapy administered. Some studies suggest that the presence of heterologous mesenchymal components, particularly chondrosarcomatous differentiation, may be associated with a comparatively more favorable outcome, although data remain limited and sometimes conflicting [19,20]. Therefore, early diagnosis and optimal cytoreductive surgery, when feasible, remain critical components of management.

## Conclusions

Ovarian carcinosarcoma with heterologous chondrosarcomatous differentiation is a rare and diagnostically challenging malignancy. This case highlights the importance of integrating clinical, radiological, histopathological, special staining, and immunohistochemical findings to establish an accurate diagnosis. The presence of massive ascites, diffuse peritoneal carcinomatosis, and a biphasic tumor pattern should raise suspicion for this uncommon entity. Reporting additional cases with heterologous differentiation may contribute to a better understanding of the clinicopathological spectrum of ovarian carcinosarcoma and improve diagnostic recognition. As the patient is currently undergoing chemotherapy, treatment response and long-term clinical outcome remain to be determined.
